# Climate Cognition Crisis: Urgent Call to Address Air Pollution and Extreme Heat Impacts in Learning Environments of Children in South and Southeast Asia

**DOI:** 10.21315/mjms-08-2025-s02

**Published:** 2025-10-31

**Authors:** Pooja Swami Sahni, Surat Dewan, Manya Sachdeva, Nayomi Ranathunga, Widad Fadhullah, Jafri Malin Abdullah, Tusar Roy, Bidhan Pal, Shoukat Baloch, Bikash Adhikari, Burcin Ikiz, Jyoti Mishra

**Affiliations:** 1 Indian Institute of Technology, Delhi, India; 2 Department of Applied Science, Technical College, Dayalbagh Educational Institute, Dayalbagh, Agra, Uttar Pradesh, India; 3 Jindal School of Public Health and Human Development, O P Jindal Global University, Sonipat, Haryana, India; 4 Department of Physiology, Faculty of Medicine, Wayamba University of Sri Lanka, Kuliyapitiya, Sri Lanka; 5 Environmental Technology Section, School of Industrial Technology, Universiti Sains Malaysia, Pulau Pinang, Malaysia; 6 Department of Neurosciences, School of Medical Sciences and Universiti Sains Malaysia Specialist Hospital, Universiti Sains Malaysia, Health Campus, Kelantan, Malaysia; 7 Brain Behaviour Cluster, Universiti Sains Malaysia Specialist Hospital and School of Medical Sciences, Universiti Sains Malaysia, Health Campus, Kelantan, Malaysia; 8 Department of Urban and Regional Planning, Khulna University of Engineering and Technology, Khulna, Bangladesh; 9 Founder and Managing Director, Probha Aurora, Dhaka, Bangladesh; 10 Sr. Public Health Specialist Department of Health, Government of Balochistan, Quetta, Pakistan; 11 Department of Wildlife and Protected Reserve, Faculty of Forestry, Agriculture and Forestry University, Chitwan, Nepal; 12 Department of Psychiatry, Stanford University, Palo Alto, California, United States; 13 NEATLabs, Department of Psychiatry, University of California, San Diego, California

**Keywords:** climate change, children, cognitive development, heatwaves, air pollution

## Abstract

The climate crisis combined with air pollution is increasingly disrupting the foundational conditions for healthy cognitive development and learning, particularly among children in vulnerable regions. In South and Southeast Asia, escalating levels of air pollution and extreme heat are synergistically threatening the neurocognitive health, educational attainment, and long-term potential of millions of children. Many schools in the region lack adequate ventilation, cooling infrastructure, and environmental monitoring, leaving children vulnerable to both acute and chronic health impacts. The review explores the effects of air pollution and extreme heat on cognitive function, behaviour, and academic performance in children. It highlights the urgent need to prioritise children’s brain health in climate adaptation, education, and public health agendas. Beyond outlining the problem, the article identifies critical gaps and opportunities for action. It stresses the need for real-time, child-centred data as a missing link for informed decision-making. The lack of public awareness and a compelling narrative around the climate cognition link is also examined as a driver of inaction. Finally, the article offers a science-driven blueprint for change, outlining a path forward that integrates neuroscience, environmental monitoring, education reform, and equity-focused policy. Understanding and responding to this “climate cognition crisis” is not only a public health and developmental imperative, but also central to building resilient, future-ready societies in South and Southeast Asia.

**Figure:**
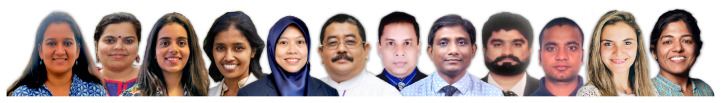

“Air pollution and climate change are not environmental issues but a public health crisis.”

## Introduction: A Silent Emergency in Schoolrooms

In the summer of 2025, students in most cities of South Asia attended classes in sweltering temperatures exceeding 40°C and inhaled air laden with PM_2.5_. The levels highly exceeded the Air Quality Guidelines given by the World Health Organization (WHO) for annual PM_2.5_ concentration (1, 2). While school closures, heat advisories, and intermittent e-learning have become accepted coping mechanisms for extreme heat and pollution episodes, a deeper, largely invisible crisis is unfolding: sustained heat and airborne toxins are compromising the cognitive health of children at critical developmental stages (3, 4).

South Asia, comprising India, Pakistan, Bangladesh, Nepal, Sri Lanka, and Bhutan, is home to over 650 million children aged 6 to 16 years. For many, daily life is dominated by higher-than-safe PM_2.5_ (including black carbon) and intensifying heatwaves (5, 6). These environmental hazards co-occur and reinforce each other. While heat promotes atmospheric inversions, where a warm air layer traps cooler air below, reversing the usual temperature decrease with altitude and blocks the vertical air mixing. This leads to the buildup of pollutants, such as smog and smoke, including black carbon, that accumulate near the ground, further intensifying warming. The resulting dual severely strains the respiratory and cardiovascular systems and uniquely affects developing brains which are highly sensitive to such stressors (7, 8).

Similarly, in Southeast Asia, where rapid urbanisation, recurrent transboundary haze, and extreme temperature events are becoming increasingly common, children face compounded risks from air pollution and heat exposure. These overlapping challenges amplify vulnerabilities in neurodevelopment and cognitive health across the region. For example, studies show that exposures related increased respiratory morbidity are now being linked to higher prevalence of anxiety, depressive symptoms, and other neurobehavioral outcomes in children (9)

Due to their rapid physical and brain development, immature immune systems, and limited ability to regulate body temperature, young school-going children are particularly susceptible to the increased exposure and negative impacts of air pollution and excessive heat that are exacerbated by climate change (10). Children, especially from disadvantaged urban environments, bear the biggest brunt, with limited access to clean indoor air, and cooling mechanisms and green spaces compound their risk. While policy responses, such as school closures during severe AQI episodes, have offered temporary relief, they inadvertently exacerbate learning inequities (11, 12). Disadvantaged children often lack access to the necessary infrastructure for remote learning, which perpetuates missed instruction, cognitive stagnation, and the widening of educational disparities (13–15).

Despite mounting evidence linking environmental stressors to dropping test scores (10) and increased ADHD-like symptoms during heatwaves (16), policy frameworks rarely address brain health outcomes. Data systems are fragmented, and public awareness about brain-level impacts remains low. Without intervention, this crisis threatens not just academic performance but the long-term neurological health of children, with profound societal and economic ramifications.

This article calls for a shift from reactive mitigation to proactive resilience. We outline a comprehensive, equity-centred scientific and policy framework designed to protect children from the cognitive consequences of extreme heat and air pollution. The proposed approach is rooted in robust real-time exposure data, neurocognitive assessments, community partnerships, and targeted policy advocacy.

## Environmental Impact: Heat, PM_2.5_, and the Developing Brain

### Synergistic Exposures and Cognitive Vulnerability

Heatwaves and PM_2.5_ do not act in isolation. Meteorological conditions, such as high pressure and temperature inversions during heatwaves, suppress pollutant dispersion, thereby increasing PM_2.5_ concentrations at ground level. Concurrently, black carbon, ultrafine PM_2.5_, a short-lived climate pollutant (SLCP), absorbs solar radiation, intensifying local warming (17, 18). Within this environmental feedback loop, children are particularly susceptible.

The physiology of children increases exposure risks. They inhale more air per body weight (6 to 12 L/min/kg in children versus about 3 L/min/kg in adults), which amplifies pollutant intake. Immature thermoregulation causes children to sweat less and overheat more quickly under thermal stress, impairing cognitive functions such as memory and attention (19). Critically, these vulnerabilities are compounded by the ongoing maturation of key brain regions. Between ages 6 and 9, the prefrontal and hippocampal areas, essential for learning and memory, undergo rapid development (20) and are especially sensitive to environmental stressors. Repeated acute exposures, such as daily commutes through polluted streets during heat spikes, can induce subclinical neuroinflammation, vascular insufficiency, and disruptions in executive brain networks (21–24). Though single-day test score drops during heat and pollution days have been documented; chronic cognitive impairments remain understudied in South Asia (25).

### Learning and Brain Loss? The Hidden Cost

Framing pandemic-era school closures simply as “learning loss” hides the real issue: the climate-affected environments. For economically marginalised children without access to air-conditioned homes or Wi-Fi, school closures can mean days, weeks, or months without proper instruction or cooling (26–28).

The true cost is cognitive, including diminished attention span, reduced working memory, and impaired cognitive function. These factors not only limit academic performance but also reduce lifetime potential. A 5-point drop in IQ across a population can lead to billions in lost productivity on a national scale (29). Collateral consequences are equally concerning, including increased anxiety, sleep problems, and socio-emotional stress during climate events. Educational systems that do not foster resilience risk perpetuating vulnerability. There is also a decline in social interactions with peers, which can negatively affect mental health and reduce opportunities for peer-driven learning and the development of social skills.

### The Present Status in South and Southeast Asia

**Figure 1:**
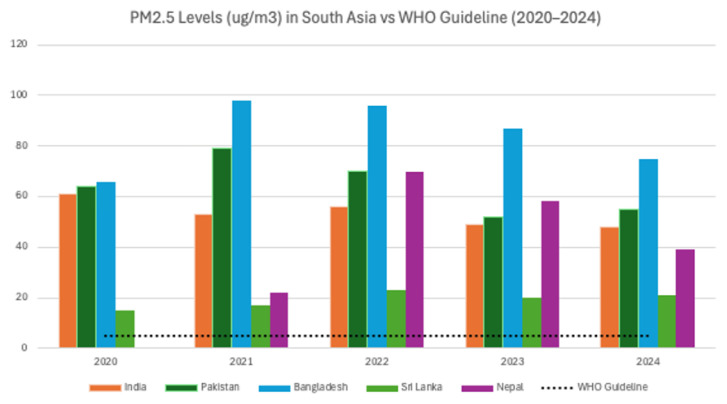
Average PM_2.5_ exposure in South Asian Countries (2020–2024)

Climate change in South and Southeast Asia is highly vulnerable due to the geographical location of the region. Heavy reliance on agriculture, limited adaptive capacity of communities, and weak emergency preparedness systems further worsen the situation. The Indo-Gangetic plains represent a critical hotspot for air pollution in the Indian subcontinent, driven by a confluence of geographical, meteorological, and human factors. Geographic barriers, such as the Himalayas, trap pollutants, while widespread agricultural practices, including crop residue burning, combined with vehicular and industrial emissions, exacerbate air quality deterioration, permeating the entire subcontinent. Furthermore, many households, particularly in rural or low-income regions, commonly use fuels such as wood, charcoal, and dung for cooking, which increases indoor air pollution and poses a significant health risk to children. Reliance on biomass and coal stoves in poorly ventilated homes leads to elevated levels of harmful particulates. Figure 1 presents the average PM_2.5_ exposure in South Asian countries between 2020 and 2024, showcasing variations across the region and changes in pollution levels over the five years. Studies have shown that in regions like India, Bangladesh, and across Southeast Asia, such exposure significantly increases the risk of acute respiratory infections, low birth weight, and childhood mortality (30, 31). However, the lack of individual monitoring makes it difficult to measure the exact pollutant intake and its potential health impact. This persistent indoor hazard further compounds the effects of outdoor air pollution and heat, intensifying the burden on children’s health. Seasonal weather patterns further intensify pollution episodes, leading to severe public health consequences, including reduced life expectancy and increased respiratory diseases. Addressing this complex challenge necessitates coordinated, region-wide policies that integrate sustainable agriculture, clean energy transitions, and robust air quality management to protect millions of vulnerable populations. Table 1 summarises recent research from the region on the rising concern that air pollution and extreme heat are negatively affecting human health and cognitive development in children.

#### India

India, with nearly 40% of its population under the age of 18, is particularly vulnerable to the impacts of environmental stressors, disasters, and climate change. Many Indian cities consistently appear in global rankings of the most polluted, according to real-time AQI monitoring and WHO reports. For example, Delhi, Ghaziabad, and Noida routinely record average AQI values above 250—classified as “very unhealthy” or “hazardous”—by both the WHO and national AQI dashboards. These levels reflect severe risks to public health due to high concentrations of particulate matter and other pollutants AQI India (5, 32). A few research studies have highlighted this growing concern over how air pollution and extreme heat in Indian cities are impairing cognitive development and academic performance in children. A 2024 study by (24) conducted in Kalinga Nagar, Odisha, assessed 30 children aged 6 to 8 years exposed to high ambient levels of PM_2.5_ and PM_10_, revealing a mean IQ of approximately 84 below the normative average, with significant deficits in attention, memory, vocabulary, mathematical ability, and spatial reasoning (24).

The CEEW Heat Risk Index report, published in 2025, identified several districts in West Bengal, including Kolkata, as highly vulnerable to night-time heat stress. In these areas, children face disrupted sleep, poor physical recovery, and increased fatigue, which lead to irritability and reduced concentration in school. The cumulative effect of extreme temperatures and poor air quality, especially in socioeconomically disadvantaged communities, highlights an urgent need for targeted interventions to safeguard the mental and academic health of children (33).

India has launched several national and regional policies to address the dual challenges of air pollution and rising urban temperatures. The National Clean Air Programme aimed to reduce concentrations of PM_2.5_ and PM_10_ by 20% to 30% by 2024, using 2017 levels as the baseline was a partial success (34). NCAP covered 131 non-attainment cities and emphasised actions such as source apportionment studies, emissions inventories, and public awareness campaigns (35, 36). The updated goal has been revised to reduce PM_10_ levels by up to 40% by 2025 to 2026. Complementing this, the National Action Plan on Climate Change includes missions such as the National Mission on Sustainable Habitat and the National Mission on Strategic Knowledge for Climate Change, which focus on mitigating the effects of urban heat and pollution (37). At the state level, State Action Plans on Climate Change have been developed by multiple states, including Rajasthan and Maharashtra, with specific components targeting heat action and pollution control (38). A pioneering example is the Heat Action Plan, initiated in Ahmedabad in 2013 and supported by the NDMA, IMD, and IIPH. This initiative has been credited with reducing heat-related mortality and has since been replicated across more than 23 states (39, 40). In Delhi-NCR, the Graded Response Action Plan (GRAP) provides a tiered approach to pollution control based on real-time air quality index levels and has shown mixed success in short-term PM reduction (41).

#### Pakistan

Pakistan, sharing its border with India and the vast Indo-Gangetic plains, faces similar challenges of air pollution and extreme heat that cross national borders. Pollutants and heatwaves affecting northern India often impact neighbouring Pakistani regions, with atmospheric transport and geographic factors driving shared episodes of hazardous PM_2.5_ and temperature extremes across this densely populated corridor People in areas dependent on agriculture, harvesting, and livestock are also affected by climate change related other issues like drought and heavy floods, causing food insecurity, malnutrition, and anaemia, further compounding the detrimental health implications for children (42, 43). The region, spanning Quetta, Turbat, Taftan, Gawadar, Hub, and Khuzdar, has been affected by increasing air pollution over the past few decades due to climate change and seasonal shifts resulting in prolonged droughts, which have led to increased dust storms and high concentrations of particulate matter (PM_10–_ and PM_2.5_) in the air. The issue is further exacerbated by rapid urbanisation, vehicle emissions, expansion of industrial and mining activities, and a lack of air quality monitoring in these regions.

There have been some steps taken at policy level for example; Pakistan joined the Paris Agreement to mitigate climate change risks and secure socio-economic development, and the government of Pakistan has put forth its National Climate Change Policy (NCCP) and passed the Pakistan Climate Change Act (PCCA, 2017) to force the implementation of the NCCP (44). Though the climate change policies for sustainable development in Pakistan are based on clean energy production, there is a need to bring more consistency and continuity in implementation, enhance institutional coordination, and address adaptation comprehensively to strengthen resilience and health, especially for children (45).

#### Bangladesh

In Bangladesh, the Climate Change and Health Promotion Unit of the Ministry of Health and Family Welfare has prepared the Health-National Adaptation Plan to strengthen the development of a climate-resilient health system. It involves professional health training, climate and health vulnerability assessments, and response development for vulnerable populations, including children (46). Although considerable research has been conducted on the respiratory effects of air pollution, rigorous assessments of its impact on cognitive function remain scarce. The absence of personal exposure monitoring makes it difficult to accurately measure individual pollutant intake, limiting our understanding of how air pollution affects brain health on a personal level. This gap highlights the urgent need for comprehensive studies that include detailed exposure tracking and cognitive evaluations to understand better and address these health risks.

#### Sri Lanka

Sri Lanka shares regional similarities with India, Bangladesh, and Pakistan in facing significant challenges from air pollution and rising heatwaves; however, its island geography results in distinct atmospheric dynamics, with coastal influences and less transboundary pollution compared to the Indo-Gangetic plains. While urban centres experience episodic spikes in air pollution, the predominance of solid biomass fuel use in rural Sri Lankan households makes indoor air pollution a more critical public health concern. Heatwave impacts, although increasing, manifest differently due to varied climatic and land use patterns (47).

Climate health impact in terms of extreme cognitive development is minimally assessed in the Sri Lankan context. The impact of indoor air pollution due to solid fuel combustion on childhood development and growth has been shown to be a cumulative, harmful effect on young children living in households that use solid fuel for cooking (48, 49). As a country with 74% of households using biomass fuel as their primary energy source, the impact on young children living in those polluted environments could be significantly higher than expected (50). More than half of the estimated child deaths were attributed to indoor air pollution, with a total of 55,300 annual deaths in adults. Adding to that, deaths due to rising heat are also predicted to increase exponentially if the greenhouse gas emissions continue to increase at current rates (50). The estimated financial loss due to premature deaths caused by air pollution is USD 2.45 billion (50). The exact impact on cognitive health due to air pollution and extreme heat in the Sri Lankan population is not yet estimated.

#### Other South Asian Countries: Nepal, Bhutan, and the Maldives

**Figure 2:**
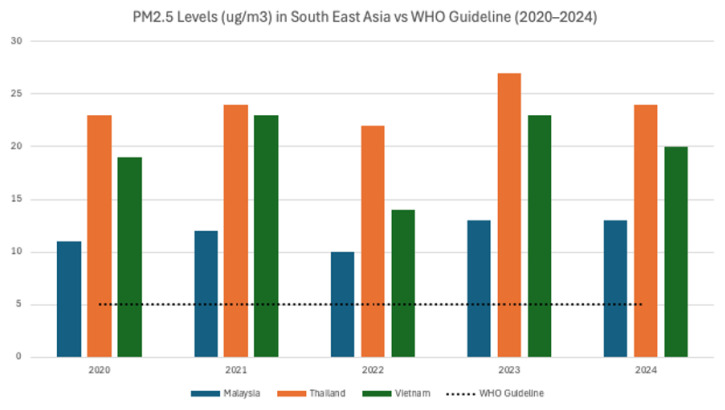
Average PM_2.5_ exposure in Southeast Asian Countries (2020–2024) Source: AQI.in

Very few studies from Nepal, Bhutan, and the Maldives have documented the adverse impacts of extreme heat and air pollution. Figure 2 illustrates the average PM_2.5_ exposure levels across Southeast Asian countries from 2020 to 2024. Nepal experienced elevated levels of fine particulate matter (PM_2.5_), particularly in the Kathmandu Valley and Terai region, which contributed to respiratory and cardiovascular diseases (56). Anobha Gurung’s pioneering research has revealed a stark reality: despite alarmingly high levels of ambient and personal air pollution in Nepal, such as average PM_2.5_ exposure of 51.2 μg/m^3^ among traffic police in Kathmandu, with hourly peaks exceeding 500 μg/m^3^, there remains a surprising scarcity of robust health-focused research in the country. Her 2013 systematic review found that only 23 out of 89 studies linked air pollution to health outcomes, with most relying on indirect exposure assessments, short study durations, and small sample sizes, focusing primarily on respiratory effects while neglecting broader outcomes, such as cardiovascular and cognitive health (56). More recent studies have begun to explore exposure in children: one investigation in southern Nepal measured personal exposure among schoolchildren aged 7 to 9 years and reported extremely high 24-hour average PM concentrations around 168 μg/m^3^ in homes, schools, and outdoor settings, far exceeding both national and WHO standards (63–65). Another biomarker study conducted in Bhaktapur examined toddlers aged 18 to 23 months and found that those from households using biomass fuels exhibited significantly shorter leukocyte telomere lengths, a potential indicator of accelerated ageing and long-term developmental risks (57). While these studies provide valuable insights into exposure and physiological effects, they do not directly assess cognitive, motor, or neurodevelopmental outcomes. This highlights a critical research gap in Nepal: the urgent need to investigate how chronic air pollution, both indoor and ambient, affects the cognitive development of children, including areas such as memory, attention, learning capacity, and behavioural health.

Concurrently, more frequent and intense heatwaves exacerbate heat-related illnesses and are associated with adverse impacts on mental wellbeing and cognitive performance (66). Bhutan experiences rising air pollution due to urbanisation and biomass fuel use, which intensifies respiratory morbidity and possibly heightens risks for anxiety and mood disorders, while extreme heat events, though less frequent, pose emerging threats to physical and cognitive health (67, 68). In the Maldives, despite relatively lower baseline air pollution, seasonal transboundary haze episodes and escalating heatwaves increase heat-related illnesses and disrupt sleep and cognitive functions, particularly in children and the elderly; however, data on long-term neurocognitive consequences remain scarce (69, 70).

#### South East Asia – Malaysia, Indonesia, Vietnam, and Thailand

Although these heat waves tend to be less severe than those observed in South Asia, they still present considerable risks to public health, agriculture, and vulnerable communities. Despite growing concern, there remains a paucity of region-specific studies systematically documenting the health impacts of combined air pollution and extreme heat exposure. Available evidence indicates serious repercussions for the health of children; for example, studies in urban environments such as Bangkok, Thailand, have shown that children are exposed to elevated levels of genotoxic pollutants, including polycyclic aromatic hydrocarbons and benzene, potentially raising their risk for DNA damage and related diseases (71).

In Malaysia, the growing impacts of climate change are increasingly visible through shifting rainfall patterns, rising temperatures, and intensifying floods (72). One such case was the Sungai Kim Kim incident. The Sungai Kim Kim disaster exposed the severe threat of toxic air pollution of benzene, toluene, xylene, and acrylonitrile, sending toxic fumes into the air and causing over 2,700 people, many of them school children, to suffer breathing difficulties, dizziness, and vomiting (73). Hotter days, still air, and heavy rains can cause toxic gases like benzene, toluene, and acrylonitrile to linger in the air longer or spread further into water.

Urbanisation contributes to air and water pollution, intensifying the urban heat island effect and raising local temperatures (74). Airborne fine particulate matter (PM_2.5_) from traffic, open burning, and industry has been linked to poorer respiratory and immune function, increasing vulnerability to severe dengue (60). In Klang Valley, sulphur dioxide and ozone are linked to higher respiratory admissions among children, while in Kuching, PM_10_ is a major concern (61). Heat exposure in Malacca’s schools has caused thermal discomfort, eye irritation, and blurred vision among students (59). A gap remains in linking acute physical impacts to longer-term neurodevelopmental outcomes; comprehensive research is urgently needed to understand how chronic exposure to heat and air pollution in Malaysian urban settings affects cognitive health and learning trajectories in children.

The interconnected threats demand urgent, child-focused adaptation and mitigation strategies. There is a need for a holistic response that seamlessly bridges immediate public health interventions with long-term climate resilience measures to address both physical and cognitive risks facing children. Public health policy, community engagement, and resilient infrastructure must work together to protect children’s health and learning environments. The National Climate Change Policy 2.0 (2024) recognises that rising temperatures worsen vulnerabilities to dengue, disrupt food, water, hygiene, and sanitation systems, and disproportionately affect women and children (75). To safeguard the next generation, Malaysia must embed a neuroclimate framework in policy and education, building not only environmental resilience but also cognitive resilience in children.

## Gaps and the Opportunity

Although emerging evidence from some of the South Asian and Southeast Asian countries suggests that extreme heat and air pollution negatively affect children’s cognitive health, impacting memory, attention span, and learning ability, this issue remains insufficiently studied and poorly understood. The region lacks a unified framework for prioritising child-centred climate–health interventions. Within countries, disparities are stark: urban poor and rural children are more likely to attend overcrowded or poorly ventilated schools, with little access to cooling, clean water, or green spaces that mitigate environmental stress. The absence of regionally coordinated efforts exacerbates inequities, allowing climate-related harms to accumulate silently in the very populations least equipped to buffer them.

Some of the several critical challenges, barriers, and gaps in addressing the cognitive health impacts of heat and PM pollution on children are described in the following sections.

### Real-time, Child-Cantered Data: A Missing Link

One of the foremost issues is the lack of child-specific health data. There are very few longitudinal or epidemiological studies that examine the effects of chronic exposure to PM_10_, PM_2.5_, and extreme heat on neurodevelopment, memory, attention, or academic performance in children (24, 76). Moreover, existing climate assessments are from very few countries in the region. The data reported in the studies mostly rely on satellite and station-based ambient averages, masking local variability in schools, homes, or playgrounds (16, 77, 78). Personal exposure, especially in microclimates such as classrooms and streets, is critically undercharacterised. The lack of neurocognitive endpoints in climate impact studies further limits policy relevance. The regions also face a shortage of monitoring infrastructure, and air quality and temperature data are collected mostly in the city centres and seldom collected near schools, childcare centres, or homes in densely populated urban areas where children spend most of their time (36).

In most of the countries in the region, there is a critical lack of integration of child-specific climate health data into health system planning. In India, climate health data systems overwhelmingly prioritise adult populations, with child-specific indicators, such as neurodevelopmental outcomes, air pollution exposure in school environments, and heat-related morbidity, being poorly represented in major national surveillance platforms, including HMIS and NFHS. For example, the National Family Health Survey (NFHS-5) captures broad health statistics but lacks granular climate-related child health metrics, and the Health Management Information System (HMIS) reports on general morbidity without regularly tracking paediatric impacts of extreme weather events or pollution (79). Consequently, the real-time assessment of climate-linked risks to children, such as spikes in respiratory illnesses during Delhi’s air pollution episodes or increased heat stress-related hospitalisations in urban centres, remains fragmented and underreported. In Bangladesh, only about 25% of operational plans fully incorporate climate change considerations, with a limited focus on the needs of children and significant research gaps in child malnutrition, mental health, and climate-induced migration (80, 81). In Pakistan, key data platforms like NADRA, HMIS, and DHIS provide limited climate-related child health information, particularly for marginalised groups such as girls and children with disabilities. Despite frequent climate disasters, such as heatwaves and floods, current data systems are adult-centred and fail to capture real-time, child-focused vulnerabilities, particularly regarding air pollution and extreme heat, which undermines effective climate response and social protection measures (82). In Malaysia, climate data systems lack unified national platforms to track air quality and extreme heat events, while economic and emissions inventories overlook key pollutants beyond CO_2_, including those driving poor air quality (83). Integrated modelling of interconnected hazards, such as droughts, floods, and heatwaves, remains underdeveloped (84).

Across the region, urban schools situated in low-income neighbourhoods or informal settlements are reported to face considerable infrastructural deficits, including inadequate ventilation, limited cooling systems, and an absence of green buffers (85, 86). These deficiencies collectively heighten children’s exposure to environmental hazards such as heat and air pollution, placing vulnerable populations at even greater risk (87).

From a research and innovation standpoint, a critical void exists in interdisciplinary studies that link air pollution and heat stressors to neurodevelopmental outcomes in children (3). Technological solutions such as wearable exposure monitors or school-based early warning systems are rarely implemented or systematically evaluated in this context.

### Fragmented Governance and Policy Inertia

The education, environment, and health sectors often operate in silos, resulting in fragmented efforts that fail to address the interconnected impacts of climate change on children’s wellbeing. There is a lack of regional prioritisation for climate health for children, and even within countries, the issues of climate change, pollution, and brain health are not holistically captured in policy.

In India, the Graded Response Action Plan (GRAP) triggers school closures at AQI “Severe+” levels, without integrating cognitive outcome data or equitable compensation for marginalised students. Other countries in the region lack even that rudimentary protocol. Policy responses remain intermittent and reactive, rather than pre-emptively protective. This situation is further exacerbated by insufficient coordination among environmental, health, and education ministries (88). The absence of an integrated, multi-sectoral approach has resulted in fragmented policies and limited child-specific adaptation strategies.

Existing national policies such as the National Clean Air Programme and Heat Action Plans, do not explicitly include children as a vulnerable group, nor do they recommend targeted interventions in school environments (38). In Bangladesh, air pollution control policies, such as phasing out two-stroke engines, promoting CNG, and the CASE Project, have seen moderate success but lack a child-focused approach. Despite progress in child health indicators in Bangladesh, the vulnerability of children to climate-induced health risks remains inadequately addressed in national research and policy. In Pakistan, political instability and weak coordination among government tiers hinder the implementation of sustainable and targeted interventions, limiting progress in programmes aimed at enhancing environmental and social resilience (89). Malaysia’s NCCP 2.0 (2024) remains heavily focused on mitigation, with inadequate attention to adaptation and financing for vulnerable communities facing heat stress and pollution-related health risks. Governance is fragmented across federal, state, and local levels.

In Sri Lanka, despite having a National Adaptation Plan since 2011 and the Ministry of Health as the climate focal point, policy responses remain limited and fragmented. Key issues, such as the cognitive health of children and targeted mitigation strategies, receive insufficient attention.

Addressing these data and governance gaps is essential to protect children from the escalating threats of air pollution and extreme heat. This lack of coordination hinders the development of integrated policies and data systems needed to effectively respond to the unique vulnerabilities of children in a changing climate.

### Public Awareness: The Missing Narrative

Aside from cosmetic air quality advisories or midday heat warnings, the link between brain health and environmental stress is absent from mainstream messaging across the regions. There exists a marked lack of awareness among parents, educators, clinicians, and local authorities regarding the detrimental impacts of air pollution and heat on paediatric cognitive development. Marginalised groups like disabled or migrant children are often invisible in datasets. Furthermore, heat illness or pollution effects are perceived as transitory, not as cumulative injuries to the cognitive potential of their child, which undermines effective action (90). Additionally, sociocultural factors, nutrition, and access to cooling infrastructure, potential modifiers of cognitive risks, remain under-researched (91).

Addressing the persistent data, policy, and capacity gaps is vital for the design and implementation of targeted, evidence-based interventions, including the creation of heat-resilient educational settings and robust early warning systems. Protecting children in urban environments, especially within low-resource contexts, demands urgent, coordinated action at the intersection of climate science, public health, and education.

Multidisciplinary collaboration offers a crucial pathway to close knowledge and policy deficits, facilitating the development of effective mitigation strategies that shield children’s cognitive development from the combined impacts of air pollution and extreme heat. Advancing these efforts will require prioritising the generation of locally relevant evidence and solutions through strong intersectoral partnerships. Such an approach is essential for establishing resilient systems capable of safeguarding the health and wellbeing of the most vulnerable child populations in the face of accelerating climate challenges.

## A Science-Driven Blueprint for Change: A Way Forward

To safeguard learning environments from the escalating threats of air pollution and extreme heat, a bold, science-driven transformation is essential. This blueprint outlines an integrated, three-pronged approach. In the following sections, the authors outline a transformative framework to reimagine classrooms as climate-resilient spaces that promote cognitive health, enhance educational equity, and ensure sustainable wellbeing for future generations.

### Expanding the Frontiers of Data

A foundational pillar of this strategy could start with the creation of a multi-country, real-time geospatial-neurocognitive dataset focused on children in climate vulnerable regions. By leveraging advanced, easy-to-use neuroimaging tools such as EEG, researchers can pinpoint functional brain changes directly attributable to environmental stressors like air pollution and heat. This granular understanding of longitudinal exposure-response relationships will not only set a new global standard for environmental health research but also provide policymakers with the compelling evidence needed to drive urgent action.

Such a dataset can serve as a global reference, enabling cross-country comparisons and fostering a nuanced understanding of how environmental hazards undermine cognitive development and educational outcomes. The insights gleaned will catalyse the translation of scientific findings into actionable policies, ensuring that interventions are both targeted and effective.

### Collaborative Policy Development

Achieving systemic change demands close collaboration with ministries of education, health, and environment, as well as leading public policy institutions and impacted communities. By co-designing solutions, stakeholders can ensure that interventions are contextually relevant and sustainable.

#### Data-Driven Advocacy

- Equip decision-makers with robust data on cognitive exposure-response relationships and comprehensive cost-benefit analyses, highlighting the long-term national gains, such as reduced healthcare costs and improved educational outcomes, with investments in climate-resilient infrastructure.
- Advocate for mandatory implementation of school-level heat action plans and enforceable PM_2.5_ limits to protect children’s health.

#### Structural and Regulatory Reforms

- Revise building codes to mandate standardised ventilation, air filtration, the integration of green walls, and the adoption of biophilic architectural principles, transforming schools into healthier, more adaptive environments.
- Amend regional air quality frameworks (such as GRAP) to include cognitive health thresholds as triggers for school closures, ensuring that children are shielded from acute environmental risks.
- Provide evidence-based guidelines for schools to integrate green infrastructure and implement non-disruptive measures during high pollution levels. Some of these measures could include the use of HEPA air purifiers to clean indoor air, limiting outdoor activities when pollution levels are high, distributing protective masks to vulnerable students and staff, implementing innovative solutions to increase ventilation without allowing polluted air to enter, and regularly monitoring indoor air quality in classrooms.
- Link budgetary allocations for green infrastructure directly to education funding, and establish a “Green School” certification programme with fiscal incentives to reward compliance and innovation.
- Build Cohesive Regional Networks for Change to ensure that those affected the most actively participate in steering the transformative change.
- Create grassroots networks of researchers and parent advocates, empowered by strong community ties, to shape and sustain environmental policy at the local level.
- Establish school-based networks that bring together researchers, students, teachers, and parents to create participatory platforms for ongoing monitoring and responsive feedback, ensuring that interventions remain adaptive and community-driven.

### Innovative Engagement Strategies

- Student-Led Monitoring: Encourage student participation in environmental monitoring by deploying floor-level sensors, providing real-time data that is accessible both within schools and homes, as well as online. This approach fosters environmental stewardship and scientific literacy among students.
- Accessible Communication: Launch multi-modal messaging campaigns, ranging from printed reports and WhatsApp alerts to school events and newsletters, that link environmental quality to school performance, making the science tangible for all stakeholders.
- Recognition and Incentives: Introduce “Clean-Air School” awards to honour classrooms that champion air- and heat-safe practices, motivating widespread adoption of protective behaviours.
- Policy Dialogues: Convene roundtable discussions with education and environment ministries across the region, facilitating cross-country learning and collaborative policy design.
- Research networks: scientists from various fields, such as neuroscience and cognitive science, climate science, child development, social health, and psychological science can ensure not only comprehensive system identification of issues but also work toward finding a solution that offers a holistic approach.

## From Evidence to Equity: A Closing Challenge

The suggested strategies are anchored in co-creation, where school leaders, parents, children, and civil society actively contribute at every stage. By redistributing ownership and centring equity, we aim not only to generate knowledge but also to empower communities to define safer futures.

The region stands at a crossroads. Without decisive action, we risk manufacturing a generational deficit in cognitive health and educational equity. But if we act with vision and purpose, we can reimagine classrooms as sanctuaries of resilience: cool, clean, biophilic spaces where children thrive in body and mind. Protecting health alone is not enough. Protecting the neurocognitive potential of our children to learn, solve, and innovate is the true yardstick of progress. The policy blueprints, tools, and community frameworks we are assembling provide a replicable model. It is not just science; it is solidarity.

As a first step in advancing the transformative agenda laid out in this editorial, the South and East Asia Neuro Climate group (SEANC), a regional subgroup of the Global Consortium’s Neuro Climate Working Group formed under Mailman Public Health School of Public Health, Columbia University, has been constituted as a collaborative alliance of scientists, clinicians, policy experts, and community leaders from across the region. The group is focused on advancing evidence-based research, advocacy, and solutions at the intersection of climate change, brain health, and educational equity. Through this article, the authors and all members of SEANC urge educators, healthcare providers, environmental advocates, and policymakers to collaborate in support of this initiative. It is essential to implement monitoring mechanisms, adopt pragmatic interventions, and establish child-centred policy standards. The opportunity to ensure a climate-resilient future for cognitive development is limited, yet still attainable. The wellbeing of the next generation depends on the actions we take today.

## Figures and Tables

**Table 1:** Studies conducted in different countries, detailing their focus areas, methodologies, and key findings related to air pollution and health

Country	Author	Title	Target population	Approach	Parameters studied	Health risks outlined
India	Ahmad et al. (16)	Association between ambient air pollution and attention-deficit/hyperactivity disorder (ADHD) in children: a systematic review and meta-analysis	Young children	Systematic review	Exposure to particulate matter (PM_2.5_, PM_10_, nitrogen dioxide [NO_2_])	Increased risk for ADHD
	Tomar et al. (51)	High-resolution PM_2.5_ emissions and associated health impact inequalities in an Indian district	Vulnerable populations (low SES)	The study linked microlevel emissions, pollution modelling, and health impacts in Saharanpur.	Surface PM_2.5_ concentration, DALYs	Premature deaths
Pakistan	Shi et al. (52)	Urbanization and regional air pollution across South Asian developing countries – a nationwide land use regression for ambient PM_2.5_ assessment in Pakistan	General population	Integrating transport networks, land use, local climate, geography, landscape, and satellite data	PM_2.5_ exposure	Health status and the liveability of life
Bangladesh	Kurata et al. (53)	Gender differences in associations of household and ambient air pollution with child health: evidence from household and satellite-based data in Bangladesh	Child health	Based on two datasets: DHS and Global Annual PM_2.5_ Grids	Exposure to PM_2.5_ and household use of solid fuels	Prenatal and postnatal health – social and biological impact
	Roy et al. (54)	Impact of fine particulate matter and toxic gases on the health of school children in Dhaka, Bangladesh	School children (9 to 12 years old)	Four hours in the school and outside the	PM_1.0_, PM_2.5_, and PM_10_ concentrations. NO_2_, TVOC, and CO_2_ concentrations	Overall health of children
Sri Lanka	Hamis et al. (55)	Prenatal PM_2.5_ exposure and its association with neurodevelopmental impairment in children: a narrative review	Children – gender based differences Tracking the impact of exposure to the pregnancy	Systematic review	Prenatal exposure to PM_2.5_	Symptoms of childhood neurodevelopmental impairment include language, motor, behaviour, social, memory, and cognition deficits
Sri Lanka	Ranathunga et al. (48)	Effects of indoor air pollution on the development of children under five years of age in Sri Lanka	Children	Prospective study assessed air quality levels and childhood development at early ages	Childhood exposure to PM_2.5_, CO and CO_2_	Children with more indoor air pollution at home had lower language, social, play, and motor development scores
Nepal	Gurung and Bell (56)	The state of scientific evidence on air pollution and human health in Nepal	Among traffic police in Kathmandu	Study relied on indirect exposure assessments, which were of short duration and small in sample size	PM_2.5_ exposure study and impact on cardiovascular and cognitive health	Respiratory effects rather than broader outcomes, such as cardiovascular or cognitive health
	Schwinger et al. (57)	The association between biomass fuel use for cooking and linear growth in young children in Bhaktapur, Nepal	Children aged 18 to 23 months living in the urban and peri-urban community	Randomised controlled trial	Children’s body length and biomass fuel pollution	Children in poor urban Nepali homes who cooked with biomass were slightly shorter on average compared to others
Malaysia	Academy of Sciences Malaysia (58)	Lessons from Sungai Kim Kim, Pasir Gudang	General population	Environmental sampling data (air, water, and soil) with public health surveillance data	Environmental variables (air quality, water quality), public health variables	Respiratory effects, neurological symptoms, and potential cognitive impacts
	Puteh et al. (59)	An analysis of comfortable teaching and learning environment: community response to climate change in school	School children	Cross-sectional survey and questionnaire-based assessments	Perceived teaching–learning comfort (thermal) and factors like indoor temperature and ventilation	Heat-related health effects, respiratory and air quality risks, Cognitive and learning impacts, and psychosocial stress
	Anwar et al. (60)	Factors related to air pollution and impacts on respiratory health in Malaysia	General populations	Scoping review	Air pollution variables	Respiratory health risks, immune system risks
	Sahani et al. (61)	Impacts of climate change and environmental degradation on children in Malaysia	Children	Climate, haze and air pollution epidemiology study and community survey	SO_2_, O_3_, PM_10_	SO_2_ and O_3_ were linked to higher respiratory hospitalisations, while in Kuching, only PM_10_ was associated with increased child admissions
Malaysia	UNICEF Malaysia et al. (62)	Impact of climate change on children: a Malaysian perspective	Children	Multi-method exploratory study	Climate wellbeing and health variables, education and learning variables, child health and wellbeing	Heat-related illnesses, respiratory diseases, psychosocial and mental health risks
